# Large Scale Characterization of the LC13 TCR and HLA-B8 Structural Landscape in Reaction to 172 Altered Peptide Ligands: A Molecular Dynamics Simulation Study

**DOI:** 10.1371/journal.pcbi.1003748

**Published:** 2014-08-07

**Authors:** Bernhard Knapp, James Dunbar, Charlotte M. Deane

**Affiliations:** Department of Statistics, Protein Informatics Group, University of Oxford, Oxford, United Kingdom; Baltimore, United States of America

## Abstract

The interplay between T cell receptors (TCRs) and peptides bound by major histocompatibility complexes (MHCs) is one of the most important interactions in the adaptive immune system. Several previous studies have computationally investigated their structural dynamics. On the basis of these simulations several structural and dynamical properties have been proposed as effectors of the immunogenicity. Here we present the results of a large scale Molecular Dynamics simulation study consisting of 100 ns simulations of 172 different complexes. These complexes consisted of all possible point mutations of the Epstein Barr Virus peptide FLRGRAYGL bound by HLA-B*08:01 and presented to the LC13 TCR. We compare the results of these 172 structural simulations with experimental immunogenicity data. We found that simulations with more immunogenic peptides and those with less immunogenic peptides are in fact highly similar and on average only minor differences in the hydrogen binding footprints, interface distances, and the relative orientation between the TCR chains are present. Thus our large scale data analysis shows that many previously suggested dynamical and structural properties of the TCR/peptide/MHC interface are unlikely to be conserved causal factors for peptide immunogenicity.

## Author Summary

Immune cells in the human body screen other cells for possible infections. The binding of T-cell receptors (TCR) and parts of pathogens bound by major histocompatibility complexes (MHC) is one of the activation mechanisms of the immune system. There have been many hypotheses as to when such binding will activate the immune system. In this study we performed the, to our knowledge, largest set of Molecular Dynamics simulations of TCR-MHC complexes. We performed 172 simulations each of 100 ns in length. By performing a large number of simulations we obtain insight about which structural features are frequently present in immune system activating and non-activating TCR-MHC complexes. We show that many previously suggested structural features are unlikely to be causal for the activation of the human immune system.

## Introduction

Recognition of immunogenic peptides presented by Major Histocompatibility Complex (MHC) molecules to the T-cell receptor (TCR) of T-cells is a key event in the adaptive immune response. In order to achieve this recognition process, a peptide in the MHC class I pathway will go through several processing steps [1]. First, a protein is degraded into peptide fragments by the proteosome. Second, the peptide enters the endoplasmic reticulum (ER) via the “transporter associated with antigen processing” (TAP) or alternative pathways such as Sec61 [2]. Third, a potential epitope must bind to the MHC class I molecule. Finally, this peptide/MHC (pMHC) complex is presented at the cell surface where its recognition by the complementary determining regions (CDRs) of TCRs can take place.

Predicting whether a peptide will undergo the initial steps (one and two) outlined above has been shown to have only a minor impact on the quality of T cell epitope prediction. This is probably due to the inability to accurately model these processes [3], [4]. In contrast, the prediction of the binding between peptide and MHC is well understood and frequently utilized for the prediction of potential T cell epitopes. Pan-specific peptide/MHC binding affinity prediction methods have reached coverage of almost all MHC class I [5] and class II [6] alleles. However, while the affinity prediction accuracy is high and binding affinity between peptide and MHC is a commonly used indicator for peptide immunogenicity [3] it is known that binding between peptide and MHC is necessary but not sufficient for T cell activation [7]–[13]. It is an obligatory prerequisite i.e. If a peptide does not bind or only binds very weakly to MHC, the necessary density of cell surface pMHC cannot be reached and T cell activation cannot take place. However, a peptide binding strongly to an MHC is no guarantee of T cell activation. Thus it is necessary to predict peptide immunogenicity, but this has proved far more challenging than prediction of the peptide/MHC binding affinity. This is mainly due to a limited understanding of which properties determine an MHC-binding peptide as immunogenic in contrast to peptides binding to the same MHC but being non-immunogenic [12]. The question “Which parameters are the driving force behind T cell activation?” is still a matter of frequent discussion and not understood in detail [14]. Suggested determinants range from binding affinity, association and dissociation rates, and half-life of interaction [11] to structural adjustments in the TCR/pMHC interface [10], [15], amino acid preferences [12], changes in heat capacity [16], similarity in biochemical properties [17], hydrophobicity, molecular weight, and structural patterns in the peptide [9]. There are two major hypotheses for T cell activation [11], [14]: (1) The affinity model i.e. the number of TCRs binding to pMHCs is the most important factor and (2) the half-life model i.e. the TCRs must bind to pMHC with a certain binding affinity and duration. Another proposal groups models for TCR triggering into aggregation models, conformational change models, and segregation models [18]. Despite all the advances over the last few years there is still much to learn about MHC class I restricted immune responses [19].

Methods for the prediction of peptide immunogenicity as opposed to peptide/MHC binding affinity, are rare [3] and show limited accuracy. Recently the method POPISK [9] has pioneered the field of immunogenicity predictors. However, an independent evaluation yielded almost random results for this method (AUC 0.52 and 0.49) [12]. Although it seems that some amino acids, especially large and aromatics (e.g. W, F, I), are likely to be associated with peptide immunogenicity, the predictive power of their presence is quite limited [12].

Therefore, since peptide immunogenicity is hard to explain from the peptide sequence alone research groups have investigated the spatial dynamics of (TCR)pMHC complexes computationally. Many immuno-informatics studies have used MD simulations to investigate the spatial dynamics of different systems. Experiments have included the use of the same MHC with different peptides [10], [20]–[31], different MHCs with the same peptide [20], [25], [32], [33], the same peptide/MHC complex with different TCRs [24], [27], simulations including trans-membrane regions [22], [34], peptide free simulations [29], [35], [36], steered simulations [27], [30], and single simulations [37], [38]. In most of these cases the real immunological outcome is known. Subsequently the differences between runs have been compared on the basis of typical MD simulation descriptors. For example Reboul et al. [33] performed 100 ns simulations of HLA-B*35:01-LPEP, HLA-B*35:08-LPEP, and SB27-HLA-B*35:08-LPEP. The SB27 interaction with HLA-B*35:08-LPEP induces a cytotoxic T-cell response while the interaction with HLA-B*3501-LPEP does not. In their simulations the authors find an increased flexibility of the peptide bound to HLA-B*35:01 and propose that difference to impede productive interaction with SB27 and therefore hamper cytotoxic T-cell response. In another study, Narzi et al. [20] used 400 ns MD simulations to investigate the ankylosing spondylitis-associated HLA-B*27:05 as well as the non-ankylosing spondylitis-associated HLA-B*27:09 with one viral and three self peptides. They found an increased entropy for the viral peptide presented by the disease associated MHC allele. For the same allele they find enhanced flexibility of the α_1_-helix which they hypothesize to be important for receptor binding. In complementary work Kumar et al. [25] performed 120 ns simulations of the multiple sclerosis predisposing allele DRB1*15:01 and the protective allele DRB1*16:01. Both alleles were simulated in combination with a myelin basic protein peptide as well as Epstein Barr Virus derived peptide. The predisposing allele formed a stable complex with both peptides. In contrast the protective allele did not form a stable complex with the virus peptide. In another investigation Stavrakoudis [37] simulated the same structure as used in this study. He performed a single 20 ns simulation of the wild-type peptide and found two conformational clusters in the peptide structure as well as that the TCRpMHC interface becomes increasingly solvated over simulation time. In a previous study we have used MD simulations to support experimental peptide/MHC binding affinity and T cell activation data measured by collaboration partners. In a mugwort pollen allergen model we compared core-identical 12-mer and 18-mer peptides bound by HLA-DR1*01:01 with the outcome of 20 ns MD simulations [21]. In a second study we compared the experimental results of altered versions of the 12-mer in complex with HLA-DR1*01:01 and HLA-DR1*04:01 with the outcome of 30 ns simulations [32].

In all of the studies mentioned above only a small number of simulations were run. This approach tends to be suboptimal since two MD simulations will always differ in some aspects if the simulation time is of finite length. Even two identically parameterized simulations (using the same initial seed) might produce different trajectories due to parallelisation and floating point imprecision. On this basis and the fact that a TCRpMHC system consists of roughly 8000 heavy atoms one might always be able to describe some differences between two individual simulations. This problem is made worse as the differences between simulations may be real but unrelated to the immunogenicity of the peptide. For example if an MHC with a very immunogenic peptide and an almost identical non-immunogenic peptide whose position seven is point mutated with a smaller amino acid yield differences, one might not be able to distinguish whether these differences result from a size change at position seven or from different peptide immunogenicity.

If one wants to address this issue then a frequent challenge is the choice of appropriate experimental data. This is problematic because some experimental findings may be false positives [39], [40], hard to reproduce, and/or not comparable with other experimental results [41]. These problems might be caused by unknown marginal differences in the experimental conditions, unintended human influence, or just different consumables used. On this basis the first aim for a systematic and large scale characterization of TCRpMHC interaction is to find an appropriate test set with experimental immunogenicity data. For our study we selected the data from Kjer-Nielsen et al. [42] because all data was (1) determined using the same technique, (2) the same conditions, (3) published by the same group, (4) in the same manuscript, and (5) the data set contains a sufficient number (172) of systematic experimental immunogenicity values. (6) In addition a crystal structure of exactly this complex was determined by the same group (Protein Data Bank (PDB) [43] accession code *1mi5* [42]). Kjer-Nielsen et al. performed a fine specificity analysis of LC13 cytotoxic T cell (CTL) reactivity to all possible single substitution altered peptide ligands (APLs) of the Epstein Barr Virus (EBV) peptide FLRGRAYGL bound by HLA-B*08:01. The employed assay of Kjer-Nielsen et al. is described in more detail in [44]. This yields a total of 172 experimental values (19 amino acid substitutions in 9 peptide positions and the wild-type).

To determine which and if any structural and dynamical factors are actually involved in peptide immunogenicity we performed 172 systematic 100 ns MD simulations of the TCRpMHC system described above. This study is far larger than any other previous (TCR)pMHC MD study. Our study assumes that if a structural or dynamical factor is involved in determining peptide immunogenicity it would be conserved across the peptides in our set. It is possible that different factors play different roles for every peptide. Our large scale test finds very little evidence of conserved structural and dynamical differences which could be related to peptide immunogenicity.

## Methods

### Modelling of the APLs

**Figure 1:**
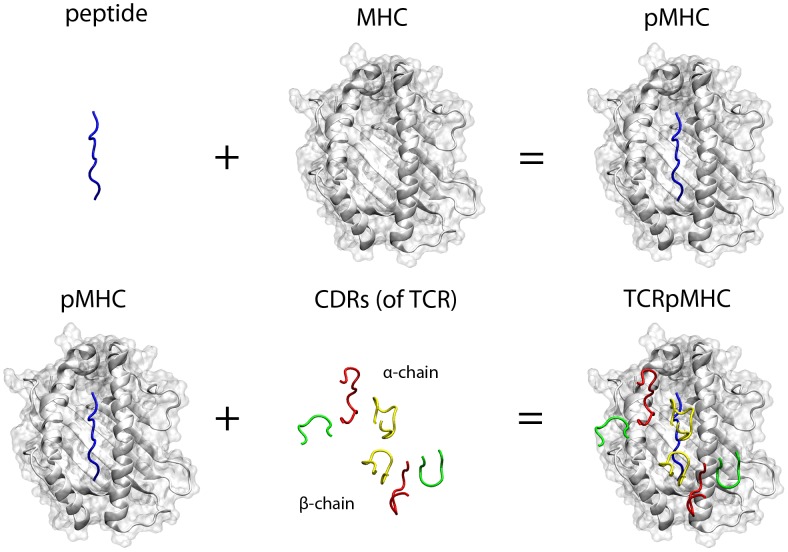
Visualisation of the individual components of the TCR/peptide/MHC interaction. Rendering is based on PDB accession code *1mi5* [42]. Blue: peptide; White cartoon and transparent surface: MHC; Red: CDR1; Green: CDR2; Yellow: CDR3. The upper CDRs belong to the TCR α-chain while the lower ones belongs to the TCR β-chain. The α_3_ region of the MHC, the β_2_ microglobulin, and the variable and constant regions of the TCR are not visualized but were also included in our study.

We used the above described crystal structure of the FLRGRAYGL peptide bound by HLA-B*08:01 and presented to the LC13 TCR (PDB accession code *1mi5*) as our basis. We modelled all possible 172 single point APLs. For each of these altered peptide ligands experimental immunogenicity data exists [42]. For this purpose we used the software SCWRL [45] via the PeptX framework [46] to replace the side-chains. We have previously shown that SCWRL is the most appropriate software in the context of peptide/MHC interactions [47], [48]. The peptide's backbone structure is relatively conserved within the same MHC allele [47] and small expected changes induced by single side-chain substitutions are accommodated by the subsequent energy minimization. The α_1–3_ regions of the MHC, the β_2_-microglobulin, as well as the variable and constant regions of the TCR were included in the models yielding 172 TCRpMHC complexes each consisting of 827 residues. One such model complex is shown in Figure 1.

### Performing the MD simulations

All MD simulations were performed using GROMACS 4 [49] and the GROMOS96 53a6 force field [50]. Each of our 172 modeled structures was immersed into a separate dodecahedronic simulation box of 3410 nm^3^ volume which was filled with ∼107,750 explicit SPC water molecules allowing for a minimum distance of 1.5 nm between box boundary and protein. Na+ and Cl− ions were also added to achieve a neutral charge and a salt concentration of 0.15 mol/liter. Each of the systems was energetically minimized using the steepest descent method and then warmed up to 310 K. Finally, we conducted MD simulations of 100 ns on each of the systems using the ARCUS cluster of the Oxford Advanced Research Computing (ARC) facility. This yields a total simulation time of 17.2 µs for our 827 residue systems.

### Methods of trajectory evaluation

Here we consider the first 10 ns of each simulation to be the initial relaxation time of the system. All analysis is based on the last 90 ns. Manual pre-inspection of the trajectories was carried out using the vmdICE plugin [51] of VMD [52]. All graphical 3D representations were rendered in VMD.

The analysis of the hydrogen bonds, solvent accessible surface area (SASA), and root mean square fluctuations (RMSF) were carried out using the GROMACS functions g_hbond, g_sas (implementing [53]), and, g_rmsf respectively. The “percent present” value of hydrogen bonds is a normalized frequency score which is zero if no hydrogen bond is present in any timeframe for this residue. It is one if one hydrogen bond is present in all frames. This score can exceed one if a residue mediates more than one hydrogen bond, as for example occurs for the anchor residues of the peptide.

The peptide/MHC binding affinities were calculated using the ligand/protein rescoring function XSCORE [54] which has been shown to be the most appropriate for structural peptide/MHC binding predictions [48]. The binding affinity between TCR and pMHC was calculated using two protein/protein rescoring functions IRAD [55] and ZRANK [56].

The relative orientation between the variable domains of the TCR, Vα and Vβ, was measured using a TCR-adapted version of the ABangle [57] methodology (shown in Figure S1). Here, the orientation is described by five angles (BA, AC1, BC1, AC2 and BC2) and a distance (DC). The length, DC, describes the distance between consistent points on the interface of the two domains. The angle BA describes a torsion angle between the variable domains of the α and β-chains. AC1 and BC1 are tilting-like angles of one domain towards the other. AC2 and BC2 describe twisting-like angles of one domain with respect to the other. The distances in the TCRpMHC interfaces were measured using the gro2mat package [58].

### Methods of comparison between more and less immunogenic simulations

Peptide immunogenicity is a continuous variable. However, to compare the characteristic features of more immunogenic simulations to those of less immunogenic simulations we have introduced a discrete split between these groups. Based on the experimental data of Kjer-Nielsen et al. [42], the 51 peptides which never induce 50% lysis make up the less immunogenic group (groupL). The 51 most immunogenic peptides were designated as the more immunogenic group (groupM). Each member of groupM induces 50% lysis using a peptide concentration of 10^−6.94^ M or less [42]. All results shown below are based on this split. This is a subset consisting of the 102 most extreme cases of our 172 simulations.

The total variation distance (tvd) was used to quantify how strongly probability distributions of the above described parameters differ between our groupL and groupM sets. The tvd is defined as:
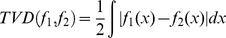
Where f_1_(x) is the first distribution normalized and f_2_(x) the second distribution normalized. Thus tvd will range between 0 and 1. A value of 0 represents perfect overlap of the distributions while a value of 1 represents no overlap. Since the tvd would yield a high value for identical means in combination with severely different variances we additionally calculate a normalized distance between the means of the distributions. This value is referred to as d/r and defined as:
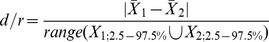
Where 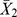 and 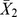 are mean value of the two distributions and the denominator is the range of the combined distributions excluding the lowest and highest 2.5%.

To further determine what values of tvd and d/r are relevant we performed permutation tests. We compared the tvd and d/r of the groups under investigation against the distribution of 2000 random assignments of the simulation trajectories to the groups. This approach is illustrated in Figure S2. If at least 90% of the permutations exhibit a smaller difference than the groups under investigation we refer to a slight difference. If at least 95% we refer to a difference, if at least 99% we refer to a strong difference.

## Results

In total we performed 172 TCRpMHC simulations of 100 ns length. In Movie S1 a movie of the wild-type simulation is shown. As described in the methods we use two subsets of simulations for the subsequent result sections. The first is made up of the 51 complexes which never induce 50% lysis (groupL). The second is made up of 51 complexes where each member induces 50% lysis using a peptide concentration of 10^−6.94^ M or less (groupM). These are the 102 most extreme cases out of our 172 simulations. Other splits were also tested and yielded similar results.

### Binding affinities

**Figure 2:**
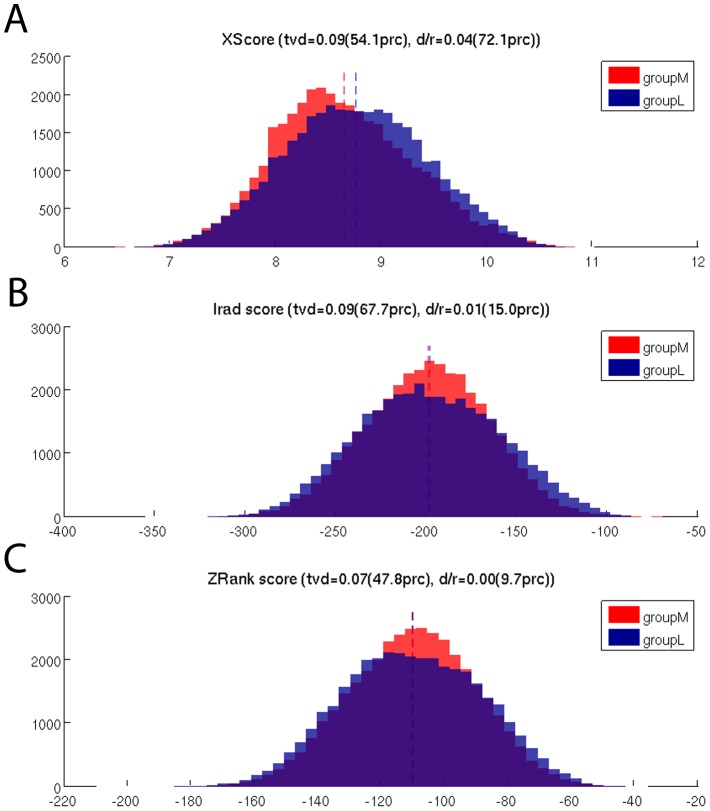
Comparison of the peptide/MHC and pMHC/TCR binding affinity distributions between groupM and groupL. (A) Binding affinity between the peptide and MHC measured by XScore. Although there are differences in the distributions, only 54% of the permutation tests show less overlap. The difference in the mean-values is larger but also not above our 90% threshold (see methods). (B) Binding affinity between the pMHC and TCR measured by Irad. (C) Binding affinity between the pMHC and TCR measured by ZRank.

The binding affinities in the TCRpMHC interface are thought to be related to peptide immunogenicty. We measured the binding affinity between peptide and MHC using XSCORE [54] on the basis of equally distributed frames extracted from the trajectories. Although the mean binding affinity of groupM is lower than that of groupL this difference is not large (Figure 2A). We also calculated the binding affinity between pMHC and TCR using both IRAD [55] and ZRANK [56], here the binding affinity distributions are also highly similar (Figure 2B and 2C) and no difference was found.

### Hydrogen bond footprints in the TCRpMHC interface

Analyzing the overall binding affinity did not yield differences between groupL and groupM. A major contributor to the binding affinity and commonly used descriptor for MD simulations are the hydrogen bond (H-bond) footprints. In our case: In which residues are H-bonds occurring most frequently during simulation and is there a difference between groupM and groupL?

**Figure 3:**
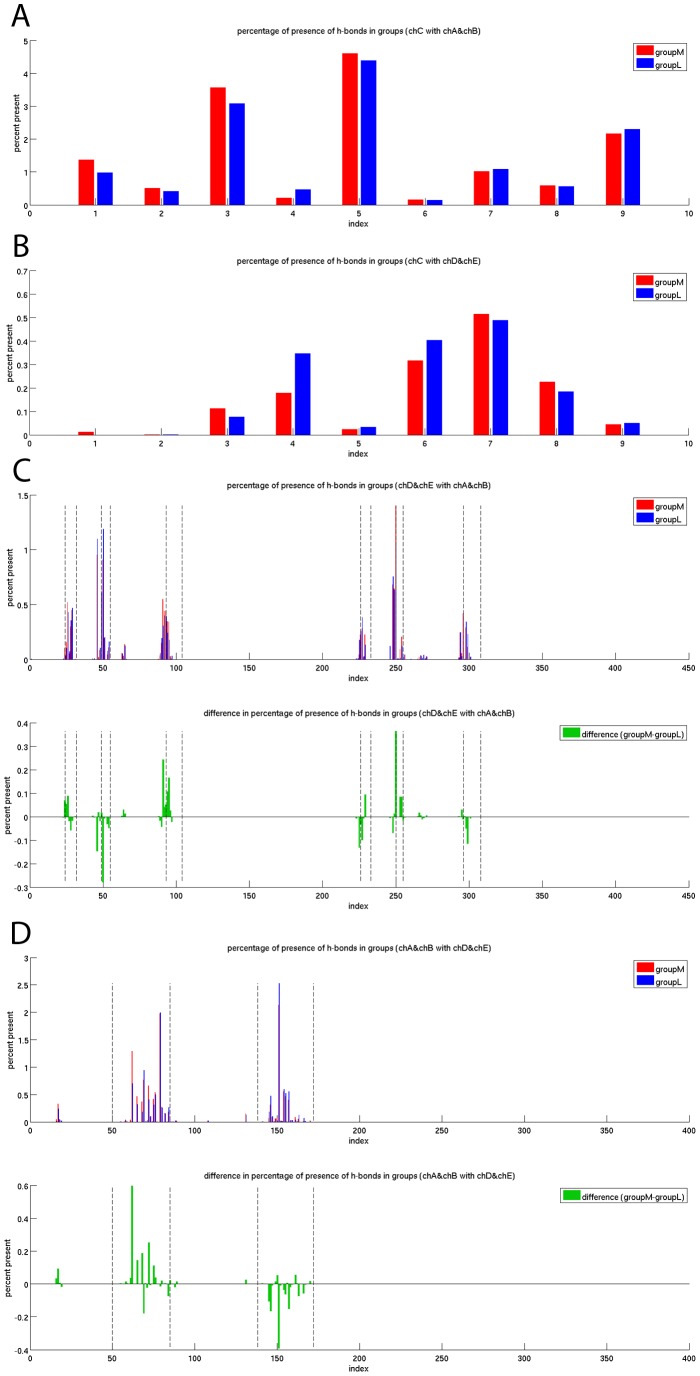
Hydrogen bond footprints of the 100 ns MD simulations of groupM and groupL. The normalized frequency of occurring H-bonds over simulation time is shown. The frequency is zero if no H-bond is present in any frame of any of the simulations of the group. The frequency is one if one H-bond is present in each frame of every simulation of the group. The value can exceed one if in average more than one H-bond is present in a residue. (A) H-bonds between the peptide and the MHC. (B) H-bonds between the peptide and the TCR. (C) H-bonds between the two chains of the TCR and the MHC. The six CDRs are marked with dashed lines. (D) H-bonds between the MHC and the two TCR chains. The helices are marked with dashed lines.

The footprint of the first frames (before MD simulation, Figure S3) differs significantly from the MD footprint (Figure 3). This highlights the importance of the dynamics of a system in contrast to static structures.

The H-bond footprint of the peptide to the MHC over simulation time recovers a key feature of the experimentally known binding profile. The anchor residues for HLA-B*08 are known to be peptide positions three, five, and nine [59]. In all three positions the number of H-bonds is higher in comparison to all other positions (Figure 3A). Additionally the number of H-bonds between the peptide and the TCR is increased around peptide position seven (Figure 3B) which has been described as the main TCR interaction site [42]. In this plot it also seems that the number of H-bonds in peptide position four is far higher in groupL. However, this can be explained by the fact that Gly is the wildtype residue for peptide position four and G4A and G4P are the two most immunogenic APLs. These three residues have no ability to form side-chain H-bonds with the TCR.

The H-bond footprints between the TCR and the MHC during the simulations revealed a preference for H-bonds to occur mainly in the CDR regions. It is experimentally known that these regions are the main interaction sites of the TCR for MHC binding [60]. However, there is no sign that more immunogenic peptides induce a different H-bond footprint in the CDRs than less immunogenic ones (Figure 3C).

A different behaviour is observed if the H-bond footprints between the MHC helices and the TCR are investigated. While groupM has an increased number of H-bonds in MHC helix 1, groupL has an increased number of H-bonds in helix 2 (Figure 3D). No mutations were introduced in the MHC helices, so the changed H-bond footprints in the MHC helices are all induced by the peptides which are adjacent to the helices but do not directly participate in the H-bonds between the helices and the TCR.

### Solvent accessible surface area of the TCRpMHC interface

**Figure 4:**
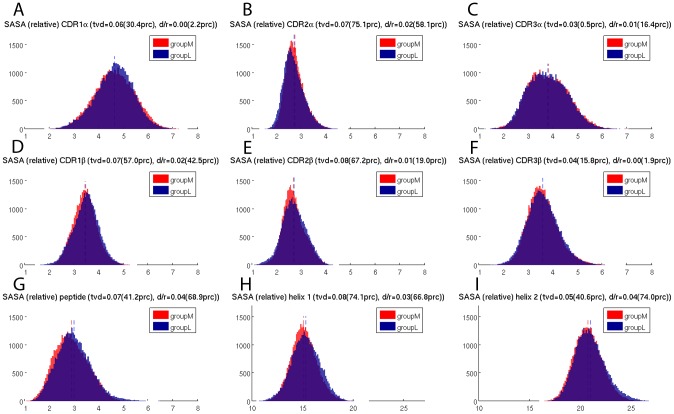
Solvent accessible surface areas (nm^2^) of the TCRpMHC interface during the 100 ns simulations. (A–F) The six CDRs of the TCR. (G) Peptide bound between MHC and TCR. (H) Helix 1 of the MHC. (I) Helix 2 of the MHC.

In addition to H-bonds, the size of the buried interface area of a binding site plays an important role in determining the mode of interaction and the binding affinity. Therefore we calculated the SASA over simulation time for the CDRs, peptide, and MHC helices. Whilst each CDR exhibits its specific distribution of SASA values there is no relevant difference between groupM and groupL and the mean values of the two groups are almost perfectly identical (Figure 4 A–F). Likewise, the distributions of the SASA values for the peptide and the MHC-helices are highly similar and no relevant difference between groupM and groupL could be found (Figure 4G–I).

### Flexibility of the TCRpMHC interface

Another feature thought to play an important role in the immunogenicity of a peptide is the flexibility of the involved interface residues. Many previous studies have hypothesized that an increased or decreased flexibility of certain components of the TCRpMHC interface is the reason for being more or less immunogenic (see discussion).

**Figure 5:**
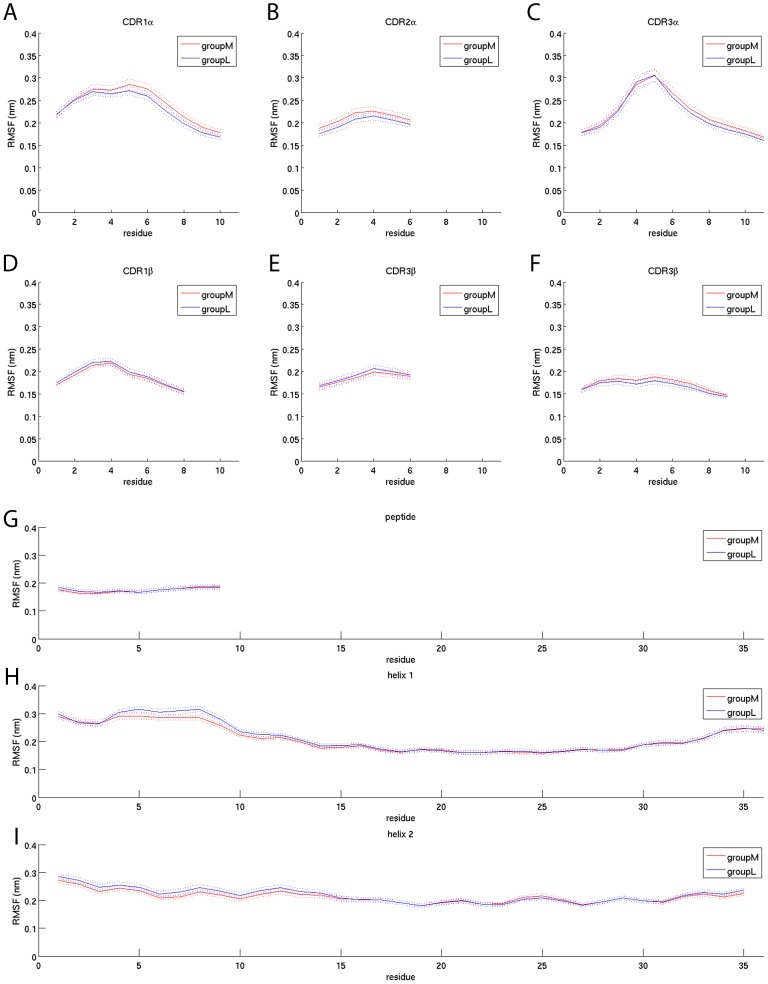
RMSF of the CDRs, peptide and MHC helices over simulation time. The solid lines indicate the mean values of groupM (red) and groupL (blue). The dotted lines are the mean +/− the standard error of the mean. The results are based on the backbone atoms only. If all atoms are taken into account the overall shape of the RMSF plots is similar, however, slightly more unstable (data not shown). (A–F) RMSF of the 6 CDRs. (G) RMSF of the peptide. (H,I) RMSF of the two MHC helices.

Therefore we calculated the RMSF of the CDRs, peptide and MHC helices over simulation time. While the RMSF of the CDRs is almost identical between groupM and groupL (Figure 5) the shapes of the RMSF curves recover a key feature of the interaction landscape. The middle part of each CDR loop is exhibiting the highest amount of flexibility which is in agreement with the notion that the CDRs are highly polymorphic and dynamic pMHC binding probes.

The RMSF values of the peptide and the MHC helices are also highly similar between groupM and groupL. We could not find an increased or decreased RMSF for either group. Furthermore the peptide shows a similar amount of flexibility in all of its residues (Figure 5G) while the helices are generally more flexible at their N- and C-terminal ends compared to their middle part (Figure 5HI).

Taken together these indicate that flexibility in the TCRpMHC interface is unlikely to be an important player in peptide immunogenicity.

### Geometry of the TCRpMHC interface

Experimental crystallographic data have shown a wide variety in the binding mode and angle between the MHC and TCR. Furthermore in-silico calculations have revealed that a state-of-the-art forcefield can reproduce these orientations [61].

**Figure 6:**
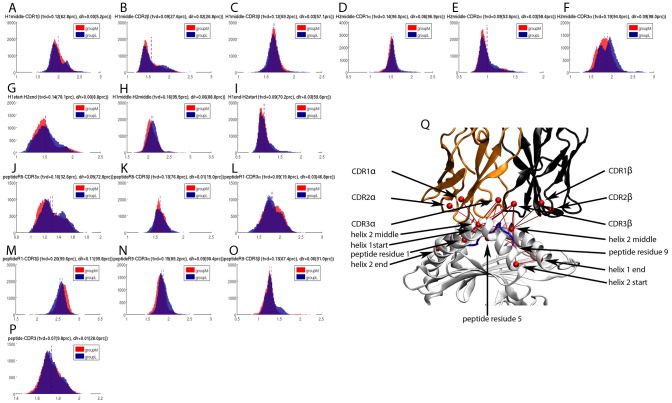
Distances in the TCRpMHC interface (nanometer) as measured over simulation time. (A–C) Distributions of the distances between the central kink residue of helix 1 and the three CDRs of the TCR β-chain (D–F) Distributions of the distances between the central kink residue of helix 2 and the three CDRs of the TCR α-chain (G–I) Distributions of the distances at the begin, middle, and end of the MHC binding groove (J,K) Distributions of the distances between the central residue of the peptide and the two CDR3s (L,M) Distributions of the distances between the first residue of the peptide and the two CDR3s (N,O) Distributions of the distances between the last residue of the peptide and the two CDR3s (P) Distribution of the distances between peptide mean and the mean of both CDR3s (Q) 3D representation of the 16 distances. Orange: TCR α-chain; black: TCR β-chain; white: MHC; blue: peptide; red: distances illustrated in A–P. The MHC helix 2 and the C-terminal end of the peptide are depicted in the foreground.

Therefore we investigated whether our groupM and groupL differ in their binding mode by calculating 16 distances in the TCRpMHC interface (Figure 6) for equally distributed individual frames of the trajectories.

The 16 distance distributions show two properties which are common to all simulations (1) no TCR detached or tilted away from any pMHC, (2) no major binding mode rearrangement took place within the interface itself.

In terms of differences between groupM and groupL we found that the distances between the central kink of MHC helix 2 are closer to CDR1a and CDR2a for more immunogenic peptides (Figure 6D,F). The distance between the middle of helix 1 and helix 2 is also decreased for groupM (Figure 6H). Only the distance between the N-terminal peptide end and CDR3b is decreased for less immunogenic peptides (Figure 6M) while the distance between the C-terminal peptide end and CDR3a as well as CDR3b is decreased for more immunogenic peptides (Figure 6N,O).

### Geometry within the TCR

**Figure 7:**
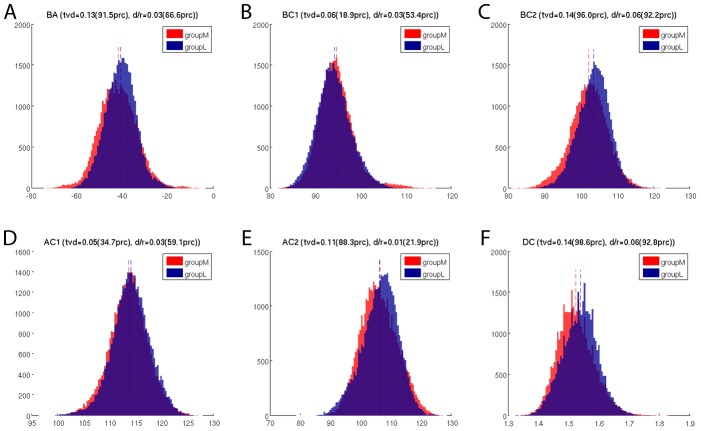
Relative orientation of the TCR chains as measured by the ABangle package. (A) BA: torsion angle between Vα and Vβ chain (B) BC1: tilting angle of Vβ (C) BC2: twisting-like angle of Vβ (D) AC1: tilting angle of Vα (E) AC2: twisting-like angle of Vα (F) DC: distance between the variable part of the α and β-chain in nanometer.

The relative orientation between the two chains of a TCR is important for their binding mode, specificity, and affinity. Therefore we investigated the relative orientation of the two TCR chains during simulation using the ABangle methodology [57]. In this way we found a slight difference in the BA torsion angle as well as a difference in the BC2 twist angle and the DC that characterises the distance between the two variable domains (Figure 7). In all three cases the mean value was smaller for groupM. These differences between the groups represent only small physical changes in orientation of the TCR chains. However, the differences that do arise suggest that groupM simulations have slightly more “open” binding site conformations best characterised by the larger (more negative) BA torsion angles.

## Discussion

### The largest systematic dataset of TCRpMHC simulations

Attempts to fully understand the interaction process between (TCRpMHC) have used MD studies (see introduction). However, all of these studies compare only a small number of simulations. For example they compare the behaviour of one MHC with two different peptides. Hence it is hard to determine if differences that are found actually relate to immunogenicity or not. To address this challenge we present, to our knowledge, the largest systematic dataset of simulations of the TCRpMHC interface. We present a dataset of 172 TCRpMHC simulations each of 100 ns.

The data shown in the results section consists of a subset of the 51 most immunogenic peptides compared to the 51 least immunogenic peptides. As we are looking for a systematic difference between more and less immunogenic peptides we showed these sets rather than all 172, as this should make such a difference easier to spot. The results of these 102 (51vs51) simulations and the full set of 172 (82vs90) simulations are highly similar (compare Figure 5 and Figure S4).

### Recovery of key features of the TCRpMHC binding landscape

Our dataset recovers several key features of the known TCRpMHC interaction landscape [60]. We show that the number of H-bonds between the experimentally known anchor amino acids of the peptide and the MHC is significantly higher than in the other peptide residues (Figure 3). Related, the number of H-bonds between the immunological hotspot of peptide position seven [42] and the TCR is significantly higher than for other peptide positions. Also the H-bonds between TCR and MHC are almost exclusively formed by the CDR regions of the TCR. Furthermore, the flexibility footprint of the CDRs (Figure 5) is in agreement with the notion that these hypervariable and flexible regions are able to scan and complement the surface of pMHCs while the framework regions around them are more rigid. No major structural defolding of TCR or MHC parts took place which is in agreement with known experimental TCRpMHC structures which have an overall conserved secondary and tertiary structure [60]. Taken together the recovered key features support the view that current state of the art MD simulations are capable of investigating the relevant dynamics of a TCRpMHC system.

### Are more immunogenic peptides weaker MHC binders?

In our dataset we found that while all the peptides are at least weak MHC binders the more immunogenic ones tend to have a marginally lower binding affinity to MHC than less immunogenic ones (Figure 2A). At first glance this seems to contradict the notion [3] that peptide/MHC binding is good indicator for a potential T cell epitope. However, even if their binding is marginally weaker, those APLs are still at least medium binders for HLA-B*08:01 and this has been shown to be sufficient to be immunogenic [62]. This tendency of more immunogenic peptides to be slightly weaker binders might be due to the previously described tendency of immunogenic peptides to have larger and more aromatic residues in the central and non-anchor positions [12]. While these residues enhance the interaction with the TCR, they may reduce, but not impair, the MHC binding affinity by increasing the entropy of the bound state.

To test whether this finding can be generalized we performed an analysis of all available experimental peptide/MHC and T cell activation data from the IEDB [63]. If all experimental matches (MHC binding affinity data and T cell activation data are available for the same peptide/MHC combination) are taken into account the correlation coefficient between T cell activation and peptide/MHC binding affinity is weakly positive (r_Pearson_ = 0.23, r_Spearman_ = 0.18). However, if only those matches are taken into account where the peptide is known to bind to the MHC (IC50<500) then the correlation drops to a slightly negative value (r_Pearson_ = −0.09, r_Spearman_ = −0.10). This would be in agreement with our finding that more immunogenic peptides, if they are at least weak binders, have a marginally lower binding affinity to MHC than less immunogenic peptides.

### More and less immunogenic peptides presented by HLA-B*08:01 have about the same binding affinity to the LC13 TCR

Often avidity between pMHC and TCR is seen as crucial for the induction of an effective immune response [11]. However, state-of-the-art computational resources are orders of magnitude away from simulating the formation of a whole immunological synapse [64]. Hence, we provide insight into the interaction of single TCRpMHC formations. On the basis of such individual interactions over 100 ns we could not find a strong difference in the binding behaviour between groupM and groupL (Figure 2B,C). Therefore the findings of our study do not support the notion that each individual more immunogenic pMHC per se necessarily binds stronger to TCRs than less immunogenic pMHCs.

### H-bond footprints of MHC helices vary between groupM and groupL

The H-bond network is commonly seen as a major player in modulating interaction landscapes. Therefore, it would not be surprising if this network is significantly altered between groupM and groupL.

A comparison of the H-bond footprint of the simulations (Figure 3) with the footprint of the static picture of the first frames (Figure S3) highlights the importance of using the 100 ns simulations. Several H-bonds are not observed in the static picture e.g. those at the N and C-terminal ends of the peptide. In contrast the first frames overestimate the number of H-bonds in many other residues. Hereby, our findings agree well with Reboul et al. who observed fluctuating and transient H-bonds in the TCRpMHC interface [33].

The H-bond footprints recover several key features of the TCRpMHC binding landscape including dominance of CDRs in the pMHC/TCR interaction, the peptide anchor residues, and the immunogenicity hotspot in peptide position seven. The H-bonds between the peptide and the MHC and TCR are very similar for the more and the less immunogenic peptide sets. However, this picture changes for the H-bond footprint between MHC and TCR. It shows a preference for more immunogenic complexes to have a higher number of H-bonds in the first MHC helix. For the second helix the picture reverses and less immunogenic complexes have a higher number of H-bonds (Figure 3D). This indicates a slightly different binding mode of the TCR to groupM and groupL. This agrees with a chemical shift mapping study that found that different TCRs can create different footprints on MHC helices [65]. Other authors described a relatively conserved CDR/helix interaction codon [66]. Some authors even hypothesize that the evolutionarily conserved kinks of MHC helices are central signaling motifs [67]. Our findings that MHC helices have different H-bond footprints with the TCR if more immunogenic peptides are present further supports the importance of MHC helices for immunogenicity of a pMHC complex.

### SASA values of CDRs, peptide, and MHC helices do not differ between groupM and groupL

The closeness of a binding interface is an important property of the mode of interaction. This closeness is often reflected in the solvent accessible surface area of interface components. For example Stavrakoudis et al. [37] reported an increased solvation of the LC13/FLRGRAYGL/HLA-B*08:01 interface, Madura et al. observed solvation as important for peptide specificity [68], and Laimou et al. [23] found that 3G of the immunodominant myelin basic protein (MBP) peptide presented by I-Au is more solvent exposed as the analogues 4A and 4Y.

From our data (Figure 4) it can be seen that the solvation of the interface changes over time and that different CDRs exhibit different distributions of their SASA values. For example, CDR1α and CDR3α show a broader distribution of their SASA values while CDR2α has rather conserved values. The SASA distributions for the peptides of groupM and groupL are both slightly positively skewed and almost normally distributed. Furthermore the first MHC helix has on average a considerably lower solvent accessible surface area than the second helix. However, while there are strong differences in the solvation of the individual parts of the TCRpMHC interface, we found no evidence that an increased/decreased solvation of any part of the interface takes place for groupM in contrast to groupL. This is surprising because if TCR/pMHC binding affinity is an important determinant of peptide immunogenicity one might expect the interface solvation to be affected. For example, MMPBSA [69] free energy calculation methods for the TCR/pMHC interface [70] contain solvation as an important term.

### RMSF values of CDRs, peptide, and MHC helices do not differ between groupM and groupL

Often authors of MD papers hypothesize that an increased or decreased flexibility of certain parts of the TCRpMHC interface is the underlying principle discriminating immunogenic from non-immunogenic TCRpMHC interactions. For example Narzi et al. observed an increased flexibility of the ankylosing spondylitis-associated HLA-B*27:05 in contrast to the non-associated HLA-B*27:09 [20]. Furthermore they found that the entropy of a viral peptide was increased compared to self peptides. Kumar et al [25] found the multiple sclerosis predisposing MHC allele DRB1*15:01 to form a stable complex with a MBP peptide as well as with an Epstein Barr virus peptide. In contrast, the multiple sclerosis protective allele DRB1*16:01 formed this stable complex only with the MBP peptide but not with the viral peptide. We have previously found that the flexibility of a mug pollen allergen peptide is increased if presented by the allergy predisposing HLA-DRB1*01:01 compared to the non- predisposing HLA-DRB1*04:01 [32]. Reboul et al. [33] reported a lower flexibility of a viral peptide presented by the CTL response inducing HLA-B*35:08 in contrast to the non CTL inducing HLA-B*35:01. In a previous study [10] we compared the CDR flexibility on the basis of 20 simulations of 50 ns length using the same TCRpMHC system as in the current study. In that study we also observed decreased flexibility of the CDRs of more immunogenic peptides (not statistically significant).

In the current study we do not find evidence that more immunogenic peptides induce a different flexibility in the TCRpMHC interface than less immunogenic peptides (Figure 5). All six CDRs, the MHC, and the peptide show almost identical RMSF patterns for groupM and groupL. This contradiction to previous MD studies might be explained by the considerably higher number of simulations in our current study. The large number of simulations of 100 ns each allows for counterbalancing confounding factors such as amino acid size, charge et cetera from which studies with a small number of simulations might have suffered.

### TCRpMHC interface and relative TCR chain orientation slightly differ between groupM and groupL

The geometry of the antibody binding site is modulated by the orientation between the heavy and light variable domains [71], and this mechanism has been proposed to be part of the diversification strategy of antibodies [72]. Here, we compared the orientation between the analogous domains in the TCR Vα and Vβ. We found that TCRs binding more immunogenic peptides tend to bind with a slightly more “open” TCR binding site. Furthermore it seems that the wider TCR binding site allows for smaller distances in the TCRpMHC interface. This difference in the binding interface could explain the previously observed difference in the early relaxation dynamics of this system [10] and could also be related to the altered H-bond footprint between MHC and TCR between groupM and groupL.

However, the absolute values of the differences between the groups are small. This is illustrated by the distance distributions between the variable segment of the α and β-chain (Figure 7F): the overlap of the distributions of groupM and groupL is lower than 98.6% of the overlaps of the 2000 random permutation splits. The distance between the mean values of the distributions of groupM and groupL is larger than 92.8% of the distances between the mean values of the 2000 permutation splits. This seems impressive, however, the distributions of groupM and groupL overlap with 86% and the difference in means of groupM and groupL is just 6% of the possible range. This is far from any predictive power and illustrates the difference between significance and relevance.

We conclude that these differences in the interface are in fact related to peptide immunogenicity but the changes in numbers are so small that peptide immunogenicity is unlikely to be explained exclusively on their basis.

### Use a large number of simulations to avoid false positives

MD studies of (TCR)pMHC complexes tend to describe the differences between a small set of structures. Often only two complexes are simulated and the differences found during the simulation are described. In our case this could, for example, be the simulation of the wildtype FLRGRAYGL and the non-immunogenic altered peptide ligand FLRGRAAGL. Both are bound by HLA-B*08:01 and presented to the LC13 TCR. These two simulations differ in several aspects. For example, the immunogenic wildtype peptide has, with respect to the non-immunogenic mutant Y7A: (1) dramatically more H-bonds between peptide position seven and the TCR but fewer H-bonds between peptide position six and the TCR (Figure S5); (2) a reduced solvent accessible surface area in CDR1α, CDR1β, CDR2β, CDR3β, and an increased solvent accessible surface area in CDR3α (Figure S6); (3) an increased flexibility for most of the CDRs, the peptide and helix 1 of the MHC (Figure S7). These differences could be argued as causal for the immunogenic behaviour of the wild-type peptide and non-immunogenic behaviour of the Y7A mutant. Scientists might be tempted to publish a manuscript about these “insights”. However, if we compare the data of two simulations (Figure S5,S6,S7) with the data of all simulations (Figure 3,4,5) than it turns out that such described differences between two simulations are false positives and that the observed differences are actually more related to the smaller side chain of Ala in contrast to Tyr than to the immunogenicity of the peptide.

To address this issue we presented the, to our knowledge, largest TCRpMHC MD simulation dataset which allows to determine which changes are in fact related to peptide immunogenicity and even more importantly, which changes are not related to peptide immunogenicity. We can draw these conclusions only for HLA-B*08:01 and the LC13 TCR but it is likely that other TCRpMHC interactions are not fundamentally different.

### Limitations of the current study

We present the largest set of TCRpMHC MD simulations carried out to date. However, even a data set of this size has limitations.

A run-time of 100 ns is longer than the average for studies of this type. Recently published MD studies of the (TCR)pMHC interface have an average runtime of about 48 ns (longest by Narzi et al. [20] with 400 ns of 12 pMHC complexes and shortest one by Stavrakoudis [37] with a single 10 ns simulation of exactly the TCRpMHC system as used in this study). However it may still not be long enough.

The membranes and the trans-membrane regions of TCR and MHC, as well as the co-receptors were not included in our study. To our knowledge there is just a single MD study [34] which included all these components. Due to the large system size Wan et al. were only able to perform a single 10 ns simulation. Furthermore, no direct experimental basis for these regions exists.

This study is based on TCRpMHC simulations. Therefore, on the basis of this data, we cannot rule the possibility out that some of the described determinants (RMSF, SASA, etc) are effective discriminators between more and less immunogenic peptides in the TCR unbound state i.e. MD simulations of pMHC instead of TCRpMHC.

In this work we are using a systematic approach, simulating a large number of peptides and looking for one or more descriptors that will differentiate more and less immunogenic peptides. Determinants for immunogenicity could vary for individual peptides. For one peptide the determinant of immunogenicity could be flexibility while it could be solvent accessible surface area for another one. If this were the case such determinants would be difficult to identify with certainty from simulations. If any two simulations are compared some differences will always be found (even for identical simulations with different random seeds and finite runtime) and it will be hard to know which differences if any relate to peptide immunogenicity.

### Conclusion

A large number of MD studies investigating the (TCR)/pMHC interaction have been published. On this basis several hypotheses about structural and dynamical determinants of immunogenicity were proposed. In this study we presented the, to our knowledge, largest TCRpMHC dataset consisting of 172 simulations of 100 ns length each. On the basis of this dataset we find that minor differences in the hydrogen binding footprints, interface distances, and the relative orientation between the TCR chains are present. Many previously predicted structural determinants of peptide immunogenicity are unlikely to be so. No striking differences could be found between the LC13 TCR recognizing more immunogenic peptides presented by HLA*08:01 compared to less immunogenic ones.

## Supporting Information

Figure S1 Visualisation of the TCR-adapted version of the ABangle [57]. A1 and A2 are the first and second principal components of structurally highly conserved C-α atoms (orange spheres) of the TCR α-chain. B1 and B2 are the first and second principal components of structurally highly conserved C-α atoms (black spheres) of the TCR β-chain. C is the distance between α and β-chain. Orange: TCR α-chain; Black: TCR β-chain; White: MHC, Blue: peptide; Red: ABangle vectors.
(TIF)

Figure S2 Illustration of the permutation test. We performed 2000 permutation iterations where in each iteration all simulations were randomly assigned to either group under investigation. The distributions of tvd and d/r yielded by 2000 permutations are shown in blue in (A) and (B) respectively. The 90^th^, 95^th^, and 99^th^ percentiles are marked as dash-dotted, dotted and, dashed lines. In addition the tvd and d/r of the group assignment under investigation is shown as solid red line.
(TIF)

Figure S3 Hydrogen bond footprints of the static first frames of groupM and groupL. The normalized frequency of occurring H-bonds on the basis of the first frame per simulation is shown. It can be seen that the H-bond footprint of the first frames significantly differs from the footprint of the whole 100 ns simulations (Figure 3). The presence of H-bonds is often overestimated in the single frame analysis while several infrequently occurring H-bonds are not characterized. (A) H-bonds between the peptide and the MHC. (B) H-bonds between the peptide and the TCR. (C) H-bonds between the two chains of the TCR and the MHC. The six CDRs are marked with dashed lines. (D) H-bonds between the MHC and the two TCR chains. The helices are marked with dashed lines.
(TIF)

Figure S4 RMSF of the CDRs, peptide and MHC helices of all 172 simulations. GroupM consists of 90 simulations and each peptide induces 50% lysis using a concentration of 10^−6.01^ M or less. GroupL consists of 82 simulations and none of their peptides induces 50% lysis at a concentration of 10^−6.01^ M or less. This figure corresponds to Figure 5 but shows 172 instead of 102 simulations. This (A–F) RMSF of the 6 CDRs. (G) RMSF of the peptide. (H,I) RMSF of the two MHC helices.
(TIF)

Figure S5 Hydrogen bond footprints of the 100 ns MD simulations of the wildtype peptide and the non-immunogenic mutant Y7A. This figure corresponds to Figure 3 and S4 but shows only two instead of 102 simulations.
(TIF)

Figure S6 Solvent accessible surface areas of the TCRpMHC interface of the wildtype peptide and the non-immunogenic mutant Y7A. This figure corresponds to Figure 4 but shows only two instead of 102 simulations.
(TIF)

Figure S7 RMSF of the CDRs, peptide and MHC helices of the wildtype peptide and the non-immunogenic mutant Y7A. This figure corresponds to Figure 5 but shows only two instead of 102 simulations.
(TIF)

Movie S1 Movie of the wild-type peptide presented by HLA-B*08:01 to the LC 13 TCR. White cartoon: MHC and β-2 microglobulin; Blue: peptide; Orange: TCR α-chain; Black: TCR β-chain.
(AVI)
