# Supplementary figures and images for: Large Scale Characterization of the LC13 TCR and HLA-B8 Structural Landscape in Reaction to 172 Altered Peptide Ligands: A Molecular Dynamics Simulation Study
Source: PLoS Comput Biol. 2014 Aug 7;10(8):e1003748. doi: 10.1371/journal.pcbi.1003748 (PMC4125040; doi:10.1371/journal.pcbi.1003748)

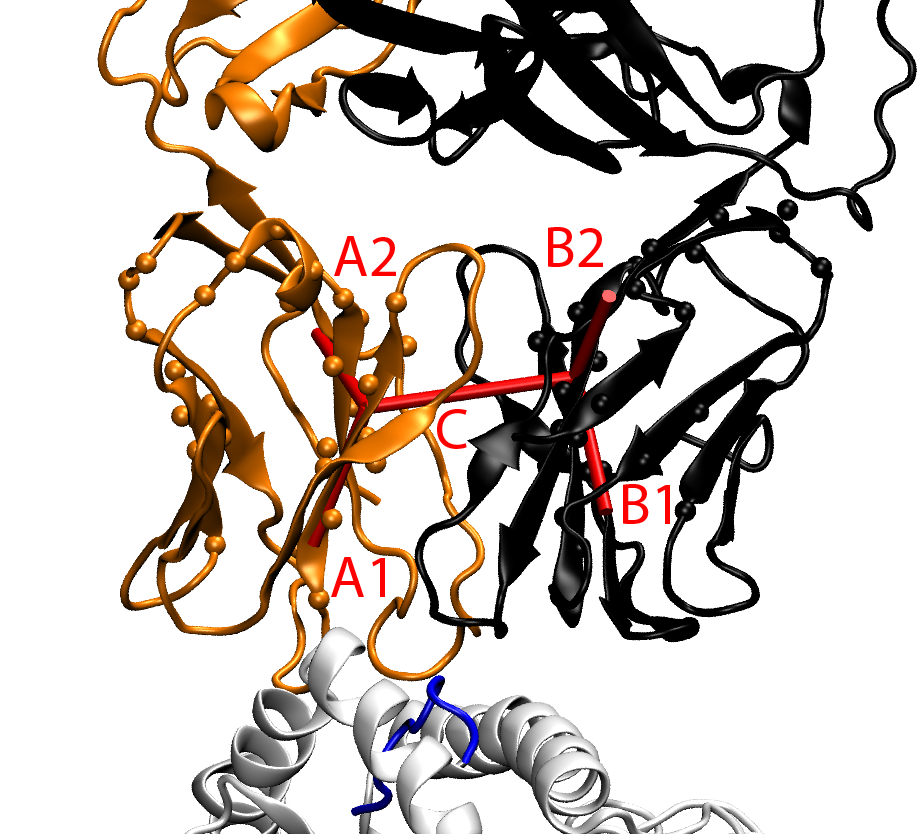

Supplement: Figure S1 — Visualisation of the TCR-adapted version of the ABangle [57]. A1 and A2 are the first and second principal components of structurally highly conserved C-α atoms (orange spheres) of the TCR α-chain. B1 and B2 are the first and second principal components of structurally highly conserved C-α atoms (black spheres) of the TCR β-chain. C is the distance between α and β-chain. Orange: TCR α-chain; Black: TCR β-chain; White: MHC, Blue: peptide; Red: ABangle vectors. (TIF) [file pcbi.1003748.s001.tif]

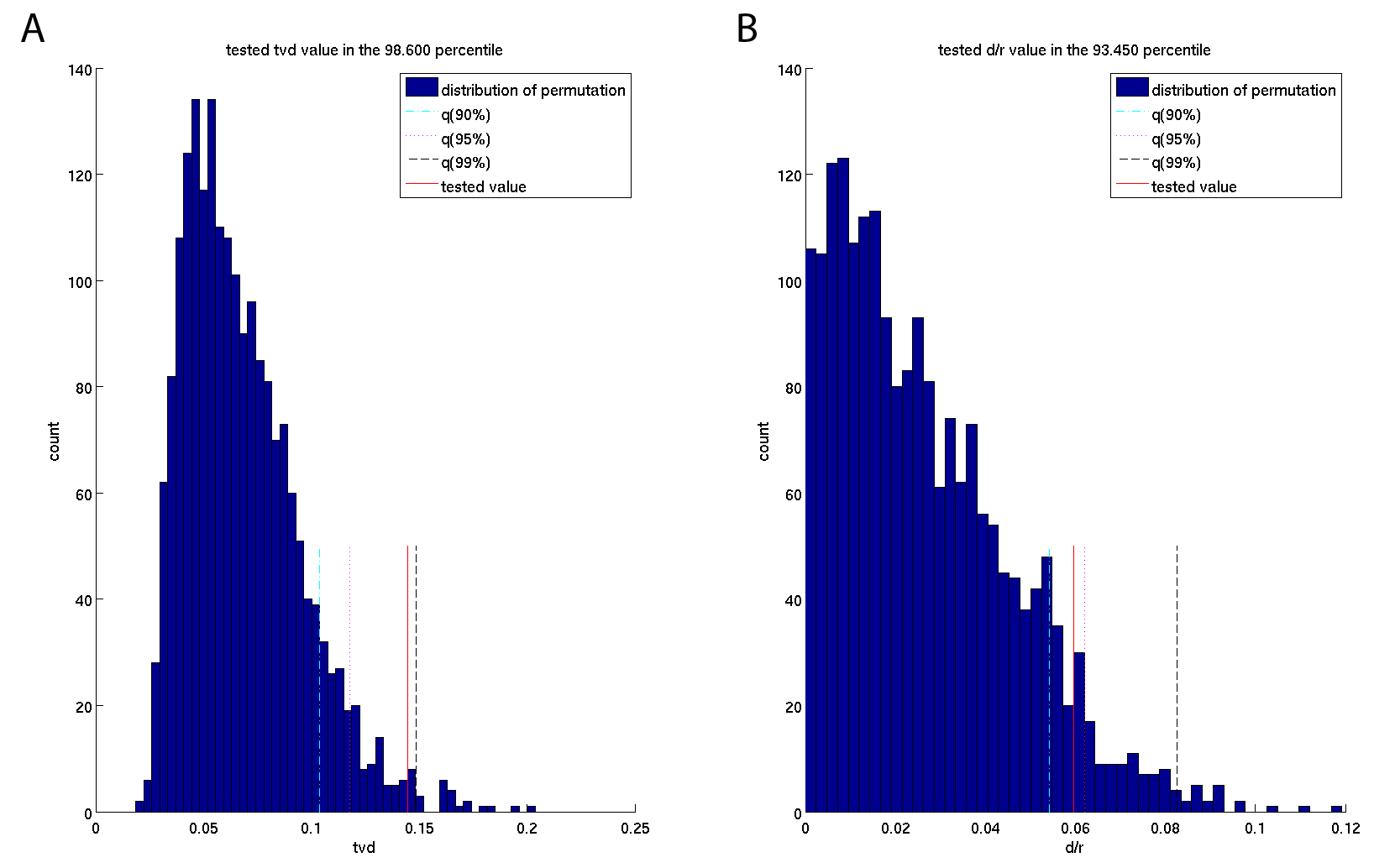

Supplement: Figure S2 — Illustration of the permutation test. We performed 2000 permutation iterations where in each iteration all simulations were randomly assigned to either group under investigation. The distributions of tvd and d/r yielded by 2000 permutations are shown in blue in (A) and (B) respectively. The 90th, 95th, and 99th percentiles are marked as dash-dotted, dotted and, dashed lines. In addition the tvd and d/r of the group assignment under investigation is shown as solid red line. (TIF) [file pcbi.1003748.s002.tif]

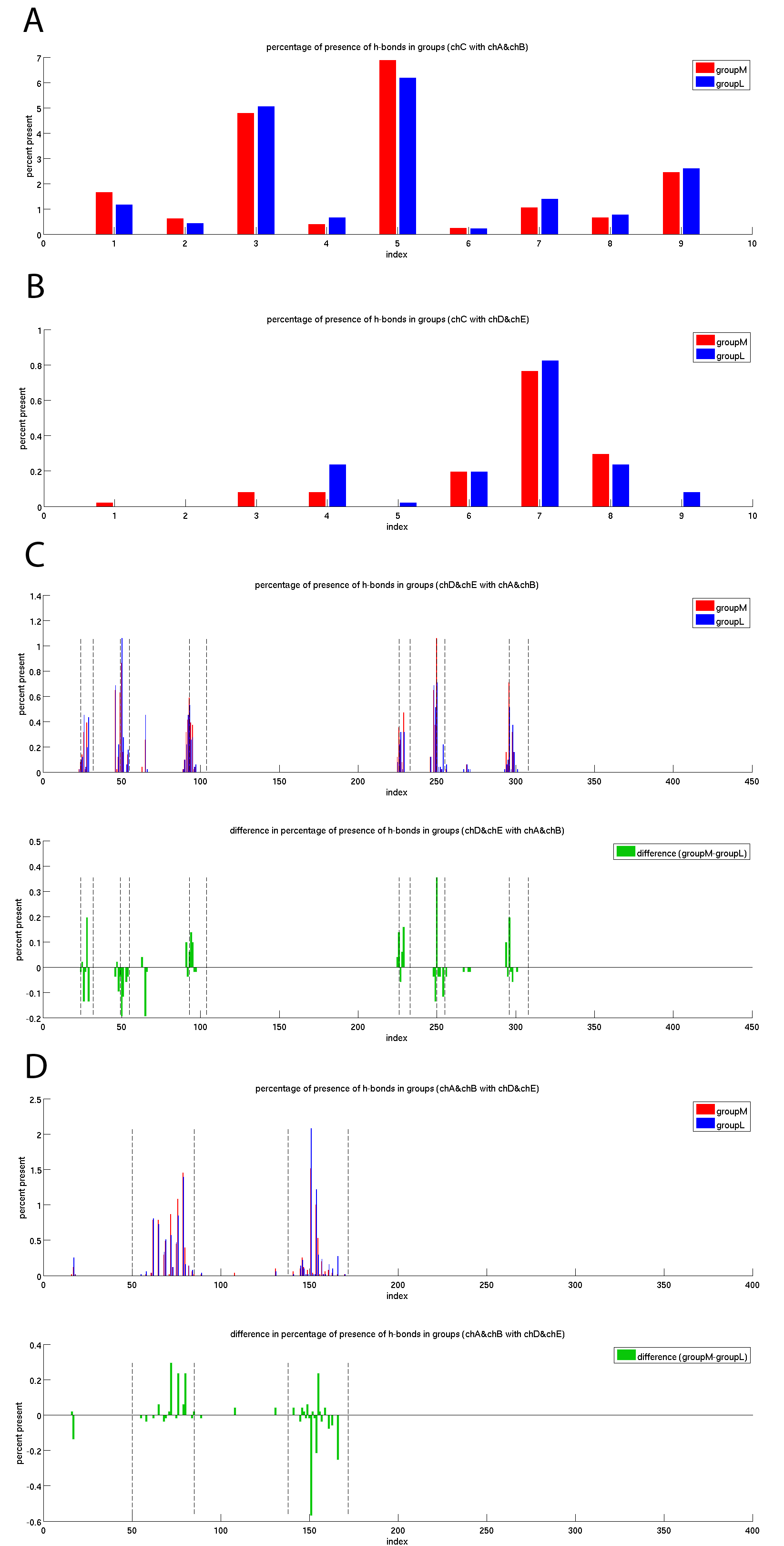

Supplement: Figure S3 — Hydrogen bond footprints of the static first frames of groupM and groupL. The normalized frequency of occurring H-bonds on the basis of the first frame per simulation is shown. It can be seen that the H-bond footprint of the first frames significantly differs from the footprint of the whole 100 ns simulations (Figure 3). The presence of H-bonds is often overestimated in the single frame analysis while several infrequently occurring H-bonds are not characterized. (A) H-bonds between the peptide and the MHC. (B) H-bonds between the peptide and the TCR. (C) H-bonds between the two chains of the TCR and the MHC. The six CDRs are marked with dashed lines. (D) H-bonds between the MHC and the two TCR chains. The helices are marked with dashed lines. (TIF) [file pcbi.1003748.s003.tif]

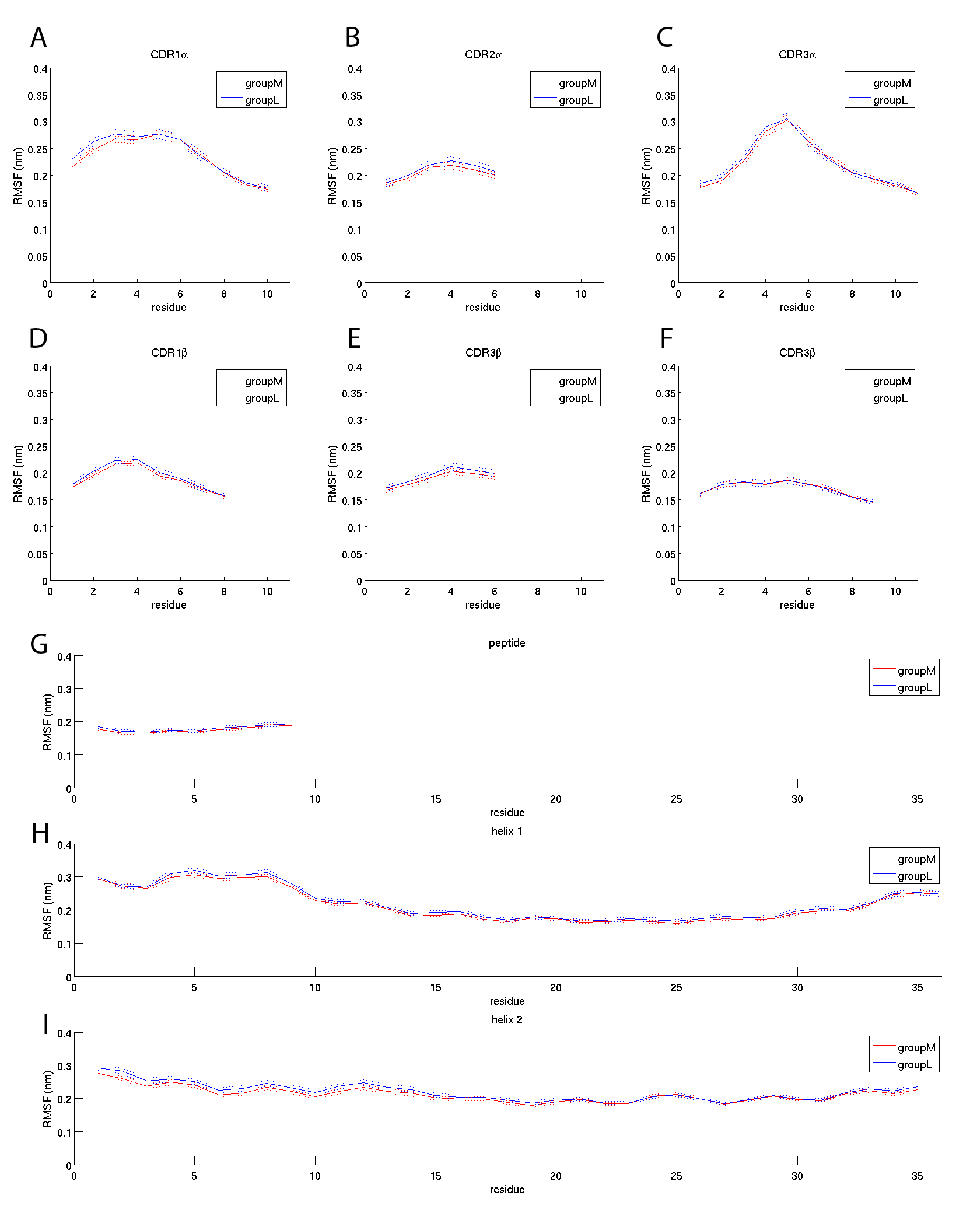

Supplement: Figure S4 — RMSF of the CDRs, peptide and MHC helices of all 172 simulations. GroupM consists of 90 simulations and each peptide induces 50% lysis using a concentration of 10−6.01 M or less. GroupL consists of 82 simulations and none of their peptides induces 50% lysis at a concentration of 10−6.01 M or less. This figure corresponds to Figure 5 but shows 172 instead of 102 simulations. This (A–F) RMSF of the 6 CDRs. (G) RMSF of the peptide. (H,I) RMSF of the two MHC helices. (TIF) [file pcbi.1003748.s004.tif]

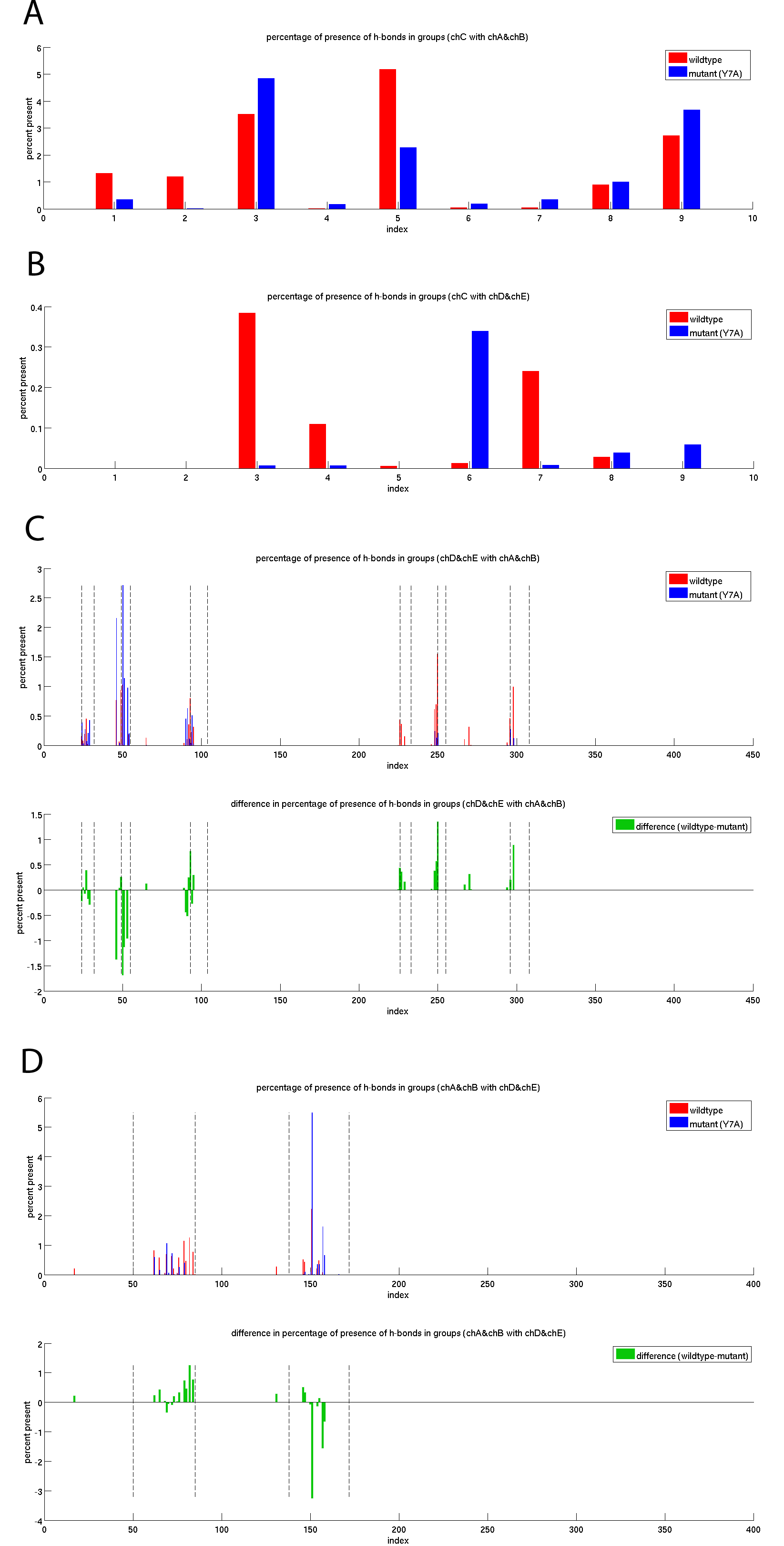

Supplement: Figure S5 — Hydrogen bond footprints of the 100 ns MD simulations of the wildtype peptide and the non-immunogenic mutant Y7A. This figure corresponds to Figure 3 and S4 but shows only two instead of 102 simulations. (TIF) [file pcbi.1003748.s005.tif]

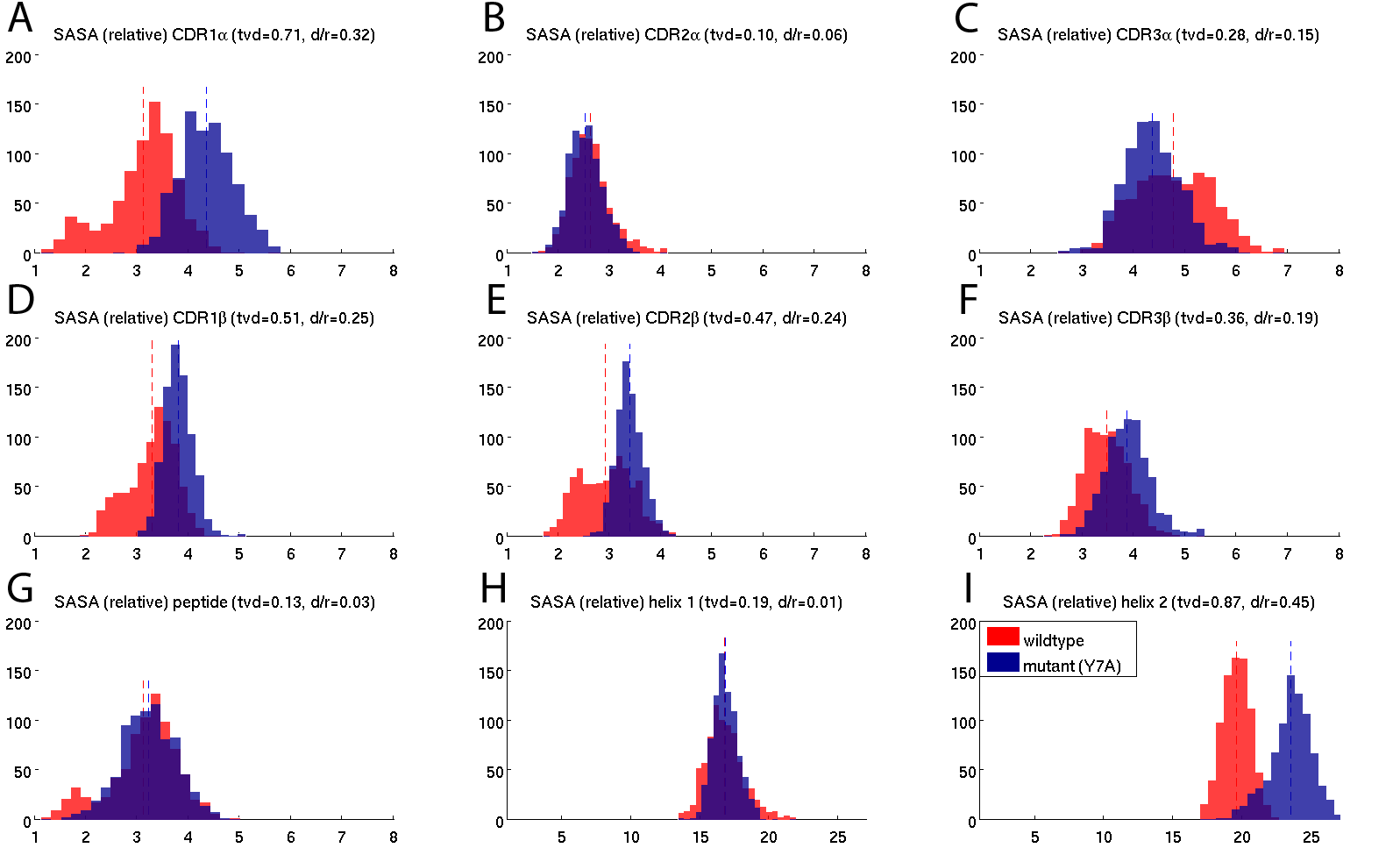

Supplement: Figure S6 — Solvent accessible surface areas of the TCRpMHC interface of the wildtype peptide and the non-immunogenic mutant Y7A. This figure corresponds to Figure 4 but shows only two instead of 102 simulations. (TIF) [file pcbi.1003748.s006.tif]

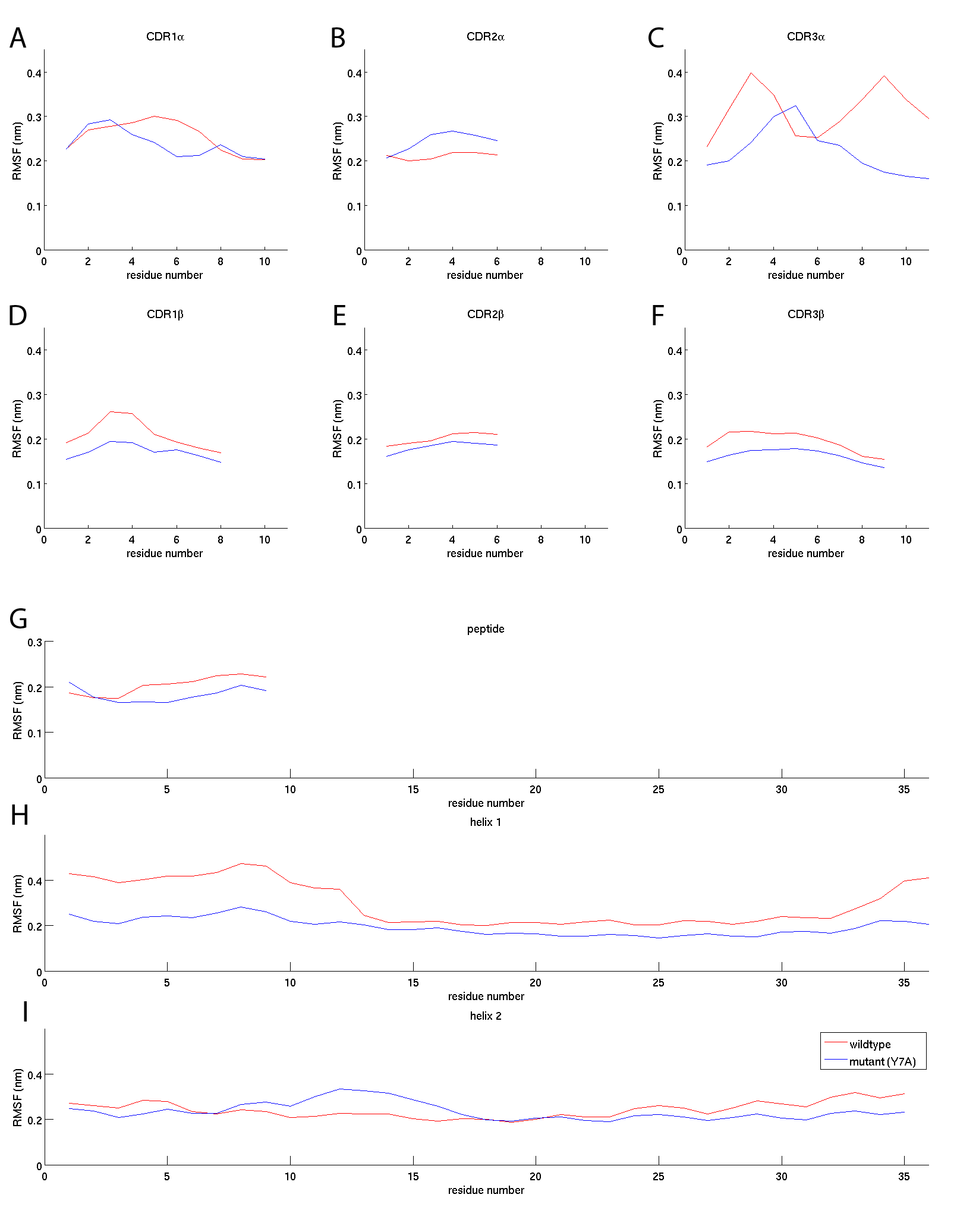

Supplement: Figure S7 — RMSF of the CDRs, peptide and MHC helices of the wildtype peptide and the non-immunogenic mutant Y7A. This figure corresponds to Figure 5 but shows only two instead of 102 simulations. (TIF) [file pcbi.1003748.s007.tif]
